# Acute-on-Chronic Liver Failure in Pregnant Patients with Chronic Hepatitis B: A Retrospective Observational Case Series Study

**DOI:** 10.1155/2020/9831687

**Published:** 2020-08-12

**Authors:** Shiwei Wang, Haofeng Xiong, Changling Luo, Hong Zhao, Ying Fan, Ting Zhang, Lili Wang, Qi Wang, Wen Xie

**Affiliations:** ^1^Department of the Liver Center, Beijing Ditan Hospital, Capital Medical University, No. 8 Jingshundong Street, Chaoyang District, Beijing 100015, China; ^2^Department of Critical Care Medicine, Beijing Ditan Hospital, Capital Medical University, No. 8 Jingshundong Street, Chaoyang District, Beijing 100015, China

## Abstract

**Background and Aims:**

Acute-on-chronic liver failure (ACLF) is common in patients with end-stage liver disease and chronic hepatitis B (CHB) or hepatitis B virus- (HBV-) related cirrhosis. To date, no uniform definition and management strategy are available for ACLF. Although a considerable number of studies on ACLF has been published, there are few reports on ACLF in pregnant women with CHB. This study retrospectively reviewed five patients who were diagnosed with ACLF during pregnancy in the past 10 years. We aimed at investigating their clinical characteristics, treatment, biochemical test results, and maternal and fetal outcomes.

**Results:**

Asthenia, anorexia, and jaundice were the main initial clinical manifestations in these patients during the second or third trimester of pregnancy. All patients received antiviral therapy. None of the pregnant women died after treatment. Patient #4 was treated with an artificial liver support system, and patients #2 and #5 underwent transfusion therapy. The acute insult in all patients was HBV DNA reactivation. Except for patient #3, who chose an actively induced vaginal delivery because of intrauterine fetal demise, the remaining four patients underwent a preterm delivery via a cesarean section. The four neonates were alive, although all were small for gestational age.

**Conclusion:**

Asthenia, anorexia, and jaundice during mid-late pregnancy should be immediately investigated. Before and during the pregnancy, hepatologists or obstetricians should actively screen pregnant women with CHB for HBV DNA status and alanine aminotransferase levels. Reactivation of HBV replication in pregnant women with CHB may lead to ACLF, especially in multiparous women. Once ACLF is diagnosed, antiviral therapy should be considered as soon as possible to protect maternal and fetal health.

## 1. Introduction

Liver diseases in pregnant women are common, and about 3–5% of pregnant women present with abnormal liver parameters [1]. The management and diagnosis of liver diseases in pregnant women are both complex and challenging. Maternal liver failure is one of the worst-case scenarios, threatening both maternal and fetal health. Liver failure presents as acute liver failure (in the absence of any preexisting liver diseases), acute-on-chronic liver failure (ACLF) (an acute deterioration of known or unknown chronic liver disease), or chronic decompensation of end-stage liver disease [2].

ACLF was first described in 1995 [3], with various definitions across different regions and working groups. In 2014, the new Asian Pacific Association for the Study of the Liver (APASL) ACLF Research Consortium presented a widely accepted definition of ACLF: an acute hepatic insult manifesting as jaundice and coagulopathy, which is complicated by ascites and/or encephalopathy, within 4 weeks in a patient with or without a diagnosis of chronic liver disease. However, the importance of extrahepatic organ failure, including lung, kidney, and circulatory system failure, has always been emphasized by Western criteria, besides liver failure alone [4].

Chronic hepatitis B virus (HBV) infection continues to be a major public health issue worldwide. While the global prevalence of hepatitis B surface antigen (HBsAg) is 3.61%, the prevalence is higher in Asian countries [5]. A meta-analysis revealed that the HBV prevalence in pregnant women in Thailand is 6.2% [6]. HBV-related ACLF (HBV-ACLF) is common in patients with end-stage liver disease and has a high mortality, especially in the Asia-Pacific and African regions. However, its clinical characteristics and progression remain unclear [7].

Unlike mother-to-child transmission (MTCT) or acute liver failure during pregnancy, little research has been conducted on HBV-ACLF in pregnant women, with scarce reports on the perinatal outcomes, clinical characteristics, and treatment modalities. Furthermore, no consensus exists on the management of this disease. This single-center case series aimed at reviewing the clinical profiles, management, treatment, and perinatal outcomes of pregnant women with HBV-ACLF.

## 2. Patients and Methods

### 2.1. Study Design

**Figure 1:**
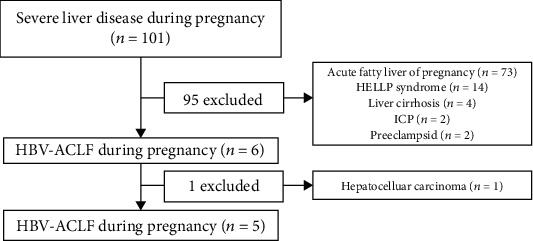
Study flow chart. HELLP: hemolysis, elevated liver enzymes, low platelet syndrome; ICP: intrahepatic cholestasis of pregnancy; HBV-ACLF: hepatitis B virus-related acute-on-chronic liver failure.

This was a retrospective review of pregnant women with ACLF who were admitted to Beijing Ditan Hospital, Capital Medical University, Beijing, China, from January 2010 to November 2018. A large number of pregnant women with liver diseases, especially liver failure was transferred from Beijing and nearby cities to this hospital. We retrospectively collected and reviewed the medical records to investigate the clinical characteristics and treatment of ACLF in this population. The study flowchart is shown in Figure 1. The study protocol conformed to the ethical guidelines of the Declaration of Helsinki and was approved by the ethical committee of Beijing Ditan Hospital. This article adheres to the applicable CONSORT (Consolidated Standards of Reporting Trials) guidelines.

### 2.2. Diagnostic Criteria of Enrolled Patients

Reactivation of HBV replication means an increase in HBV replication (a ≥2 log increase from the baseline level or the appearance of new HBV DNA to a level of ≥100 IU/mL) in a person whose HBV DNA level was previously stable or undetectable, with a level of ≥20,000 IU/mL in a person without baseline HBV DNA data [8]. Liver failure in ACLF was defined as jaundice (serum bilirubin ≥5 mg/dL [≥85 *μ*mol/L]) and coagulation (international normalized ratio [INR] ≥1.5 or prothrombin activity [PTA] ≤40%) [2]. ACLF was diagnosed according to the APASL guidelines and consensus recommendations mentioned above.

### 2.3. Data Collection

Pregnancy was diagnosed with ultrasonography. The gestational period was calculated from the initiation of amenorrhea. We extracted data on demographic characteristics; clinical symptoms; gestational period at diagnosis; delivery, parity, gravidity, and laboratory test data at admission and during the hospital stay; complications; abdominal ultrasound scans; and presence and patterns of HBV infection. Serological tests were performed using acute sera for the detection of Epstein-Barr virus, hepatitis A IgM, hepatitis E IgM, hepatitis D IgM, cytomegalovirus, herpes simplex virus, human immunodeficiency virus, and antibodies against hepatitis C and hepatitis C virus-RNA. Patients with increased serum ceruloplasmin and urinary copper levels, autoantibodies, acetaminophen usage, alcohol consumption, and sepsis were excluded. Various scores were evaluated, and all necessary parameters were available within 48 h of hospitalization.

### 2.4. Statistical Analyses

Data analysis was performed using descriptive statistics. Normally distributed data are expressed as mean ± standard deviation. Nonnormally distributed data are expressed as median and interquartile range. Data analysis was performed using SPSS version 22.0.

## 3. Results

### 3.1. Maternal Demographic Characteristics

We enrolled five pregnant women with HBV-ACLF. Three patients were infected by vertical transmission during birth, and two patients were diagnosed with HBV infection during a physical examination (one patient at age 29 years and the other patient at age 22 years; neither reported a family history of HBV infection). Abdominal ultrasound imaging confirmed that all patients were noncirrhotic. All pregnancies were singleton. The mean maternal age was 30 ± 7 years, and only one patient was <25 years old (patient #2: age 23 years). The mean period of gestation at admission was 28 + 1 weeks. Except for the youngest patient (patient #2) who was gravida 1 and para 1, the others were multigravida and para 2. Liver biopsy was not conducted in all patients during the current admission (Table 1).

### 3.2. Maternal Clinical and Biochemical Characteristics

Asthenia, anorexia, and jaundice were the main initial clinical manifestations in the patients during the second or third trimester of pregnancy. No psychiatric symptoms or hepatic encephalopathy were observed to date. As the disease progressed, the patients presented with other symptoms, including edema of the lower limbs, itchy skin, and fever (Table 2).

The serum alanine aminotransferase (ALT) and aspartate aminotransferase (AST) levels were significantly abnormal. Four women presented with an AST/ALT ratio of <1. The serum bilirubin levels were also significantly abnormal, whereas the gamma-glutamyl transpeptidase and alkaline phosphatase (ALP) levels were mildly elevated. ALP is generally unaffected by pregnancy. The ALT level during their hospitalization is shown in Supplementary Table 1. The patients tested positive for HBsAg and antibodies against hepatitis B core antigen. In three patients, the HBsAg levels were >250 IU/mL, whereas in patients #1 and #5, the HBsAg levels were 0.3 and 19.5 IU/mL, respectively. The hepatitis B e antigen (HBeAg) test was positive in three patients and negative in two patients. The mean HBV DNA was (5.3 ± 1.6) log 10 IU/L at admission (Table 3).

### 3.3. Acute Insults

A reassessment by two hepatologists revealed that the acute insult in all cases was HBV reactivation. No patient had undergone chemotherapy or immunosuppressive therapy in the past year. Except for patient #4 (baseline HBV-DNA was TND that was tested at six months before the onset of the disease), no other patients underwent any previous assessment for liver diseases. Reactivation of HBV was secondary to telbivudine (LDT) resistance in patient #4. In all other patients, HBV reactivation was spontaneous (Table 1).

### 3.4. Complications

All patients except for one experienced bacterial or fungal infections in the lungs or at least one organ, including the abdominal cavity, uterine cavity, or urinary system. Nevertheless, none of the patients developed sepsis or organ failure. Kaliopenia was observed in two patients. Only patient #5, who had the greatest number of pregnancies, experienced postpartum hemorrhage. Abdominal ultrasound imaging, conducted at least twice, did not reveal hepatocirrhosis in any patient.

### 3.5. Management and Outcomes

#### 3.5.1. Special Treatments

All patients were admitted to the intensive care unit first and subsequently transferred to the general ward when the INR decreased to <1.5, PTA exceeded 40%, and no organ failure was observed. Two patients required transfusion therapy (plasma and blood): patient #5, who was the oldest (37 years) and in the third trimester (32 weeks), experienced postpartum hemorrhage, whereas patient #2, who was the youngest (23 years) and in the third trimester (31 + 2 weeks), experienced bleeding during cesarean delivery. In patient #4, cholestasis continued to worsen despite general treatment with internal medicines; therefore, she was treated with an artificial liver support system. After two sessions, the cholestasis clearly resolved. Patients #1 and #4 were administered low doses of glucocorticoids (5 mg/day) to induce fetal lung maturation.

#### 3.5.2. Antiviral Agents

After HBV DNA detection, all patients received timely monotherapy with nucleos(t)ide analogs without considering the gestational weeks. Except for patient #4, the other patients received antiviral treatment for the first time. Patients #2 and #5 received antiviral treatment with entecavir (ETV) after delivery. The remaining patients received antiviral treatment with lamivudine (LAM), tenofovir disoproxil fumarate (TDF), and LDT before delivery (Table 4). Only patient #4 had received regular antiviral treatment. The initially administered interferon therapy was a failure; thereafter, ETV treatment was initiated, which was effective. To prepare for pregnancy, patient #4 switched from ETV to LDT under the guidance of a hepatologist, 1 year before this admission. HBV DNA was not detected at 6 months after switching (target not detected [TND]; <1.0*E* + 2 IU/mL). Considering LDT resistance, LDT was replaced with TDF during hospitalization. Two weeks later, *a* > 2 log reduction in the HBV DNA level was observed in all cases. The HBV DNA level in all patients was TND at discharge.

#### 3.5.3. Maternal and Fetal Outcomes

Fortunately, no maternal deaths occurred. All patients were in remission and did not need liver transplantation upon discharge. Except for patient #3, who chose an actively induced vaginal delivery (at 30 gestational weeks) because of fetal abnormality (multiple malformations) and intrauterine fetal demise, the remaining four patients underwent a preterm delivery via a cesarean section. The four fetuses were alive, although all were small for gestational age (SGA). The newborn of patient #2 experienced neonatal asphyxia (Table 5).

## 4. Discussion

The earliest clinical manifestations in our patients were asthenia and anorexia, which were ignored until jaundice developed. This indicates that asthenia and anorexia, especially during the mid-late trimester in pregnant women with HBV, should serve as warning signs to gynecologists, hepatologists, and the women themselves. Nausea and vomiting generally occur during the first trimester, and very few women experience these events throughout the pregnancy [9]. Most importantly, any pregnant woman suspecting pregnancy needs to undergo a pregnancy test.

A retrospective study from China reported that steroids have no value in improving nontransplant survival in patients with HBV-ACLF. However, pulmonary and opportunistic infections occurred with a higher frequency (*P* = 0.003 and *P* < 0.001, respectively) in the steroid-treated group [10]. Therefore, the necessity of steroid treatment should be adequately assessed. In our study, steroids were not used to control ACLF and other complications, and only low doses were administered for fetal lung maturation.

Liver transplantation is a highly effective intervention in specific cases; however, organ availability, timing of transplantation, and high resource use are the barriers to its more widespread application [7]. Meanwhile, Moon et al. reported 5-year graft survival rates of 70.5% and 81.0% in the ACLF and non-ACLF groups, respectively (*P* = 0.035) [11]. The benefits of liver transplantation for HBV-ACLF may not be as great as expected. Thus far, most published studies have focused on pregnancies after liver transplantation [12]. Liver transplantation in pregnant women may involve more challenges.

Granulocyte-colony stimulating factor (G-CSF) therapy has been used for ACLF. A randomized clinical trial reported that the survival rate in the G-CSF treatment group (48.1%) was significantly higher than that in the placebo group (21.4%) (*P* = 0.0181) [13]. Although still at an experimental stage, these results are encouraging [14]. It is interesting to note that G-CSF is used for a variety of diseases during pregnancy, including chronic neutropenia and pyoderma gangrenosum [15, 16]. Meanwhile, its adverse effects are mild for pregnancy. Therefore, G-CSF may be an appropriate choice for ACLF during pregnancy.

Antiviral treatment reduces the viral load, promotes a reduction in hepatocyte cell death, and subsequently improves survival outcomes [17]. Furthermore, for the reduction of MTCT, Huang et al. reported that treatment with nucleoside analogs (NAs), such as LAM and ETV, could improve the prognosis of patients with HBV-ACLF [18]. Further, they suggested that NA therapy should be applied as soon as possible. A study from India reported that a 2 log decrease in HBV DNA after 2 weeks improved the survival rate of patients with HBV-ACLF [19]. Furthermore, Yang et al. reported that an initial combination of antiviral therapy is effective in reducing the short-term fatality of HBV-ACLF [20]. Nevertheless, the survival benefits of different NAs are still uncertain in pregnant women with HBV-ACLF. The status of the fetus should also be considered. In this study, all patients received timely monotherapy without considering the gestational weeks. The American College of Gastroenterology guidelines suggest that pregnant women chronically infected with HBV and bearing a high viral load (≥200,000 U/mL or ≥6 log copies/mL) should be offered antiviral medication with TDF or LDT in the third trimester to reduce the perinatal transmission of HBV, similar to the APASL guidelines [1, 8].

The acute insults of ACLF vary according to the geography and the population and can be categorized as intrahepatic or extrahepatic. Extrahepatic insults include infections (bacterial, fungal, and parasitic) and acute variceal bleeding. In fact, 40–50% of patients with ACLF do not have an identifiable trigger [14, 21]. Intrahepatic insults include NA resistance, HBV reactivation, chemotherapy, or other immunosuppressive drugs, active alcoholism, hepatotoxic drugs, and acute flare-up of autoimmune hepatitis or Wilson disease [4]. Among the intrahepatic insults, HBV reactivation is the predominant cause in Asian countries, especially China [22–24]. A multicenter retrospective study from the United States, which spanned for nearly two centuries, observed that HBV DNA flares occurred in 9% (8/90) of the women during pregnancy and in 4% (2/48) of the women during the postpartum period [25]. HBV reactivation may be spontaneous or triggered by intensive chemotherapy/immunosuppressive therapy. Inappropriate withdrawal of nucleos(t)ide analogs, nucleos(t)ide analog resistance, or chemotherapy is common triggers. In our study, HBV reactivation was the only reason. Other acute insults were excluded according to complete medical history and laboratory findings. Nevertheless, owing to the small number of reviewed cases, it cannot be concluded that HBV reactivation is the only cause of HBV-ACLF in pregnant women. However, more attention should be paid to the fact that most of the patients did not undergo an HBV assessment before the illness. Women of childbearing age should proactively undergo assessment for HBV. Maternal HBeAg levels, HBV DNA status, and ALT levels should be checked during pregnancy [8].

Pregnancy is usually well tolerated by healthy HBV carriers and those who have chronic hepatitis B (CHB). Chronic HBV infection does not seem to be a risk factor for maternal complications such as placental abruption, preterm labor, and preeclampsia. It appears to even have a protective effect, probably owing to the impaired immune response and/or increased immune tolerance caused by the virus; however, this was only observed in the Asian population [26–28]. Some studies also reported that chronic HBV carriers have a slightly increased risk of preterm birth and low birth weight [6]. Our study supports this finding, as preterm SGA babies were born to four of the five patients.

Several studies from India have reported that ACLF has a high short-term mortality [29], with the 90-day mortality rate being 63% [19]. A prospective study from China revealed that the short-term (28/29 days) mortality of patients with HBV-ACLF was significantly higher than that of patients without HBV-ACLF [30]. In our study, although intrauterine fetal demise occurred in patient #3, none of the women died. According to the consultation findings, the intrauterine fetal demise in patient #3 may be related to chromosomal anomalies and not relate to HBV-ALCF. In women with acute liver failure, pregnancy should not be considered a poor prognostic factor [31]. More data are required on whether HBV-ACLF affects maternal or fetal outcomes. This study was unable to investigate morbidity and mortality in these patients or explain the correlation between intrauterine fetal demise and HBV-ACLF. Therefore, more in-depth studies are required on this issue.

An early and accurate prognostic scoring system is required for assessing the disease severity and optimizing the treatment [32]. The prognostic model for end-stage liver disease (MELD), sequential organ failure assessment (SOFA), and chronic liver failure-SOFA (CLIF-SOFA) were recommended by the 2014 APASL ACLF consensus [2]. MELD is limited to assessing the prognosis of ACLF, ignoring the existence of all potential extrahepatic organ failures. The CANONIC study developed the CLIF-SOFA score for European patients with acute hepatic decompensation [14, 33]. In this study, four prognostic scores were used. No obvious differences were observed (Table 6). The maternal outcomes were also good in all cases. Although our study could not assess these prognostic scores, some good-quality studies have been conducted on them [34, 35]. Very few studies have investigated whether these scoring systems are applicable to pregnant women. Biomarkers, such as cell death biomarkers (M30 antigen, M65 antigen, and high mobility group protein box 1), that can predict clinical outcomes or assess severity may serve as potential research subjects [36].

The long-term effects on women and neonates require further follow-up with respect to the correct management of ACLF. Many questions remain unanswered owing to the relative lack of data, including why HBV reactivation, resulting in ACLF, occurred in these cases; whether there are any connections between pregnancy or gravidity and perinatal outcomes; and, more importantly, whether ACLF increases the risk of MTCT and whether antiviral agents lower the obstetric complications in these patients. All these questions require high-quality research, and prospective studies with a large sample size are urgently needed.

## 5. Conclusion

Asthenia, anorexia, and jaundice during mid-late pregnancy should be immediately investigated. More importantly, pregnant women with CHB should undergo routine screening. Screening for HBV DNA status and ALT levels is recommended before and during the pregnancy. Reactivation of HBV replication in pregnant women with CHB may lead to ACLF, especially in multiparous women. Antiviral agents and premature birth should be considered when ACLF is diagnosed to protect maternal and fetal health.

## Figures and Tables

**Table 1:** Maternal characteristics.

Patient	Age (years)	Gravidity (times)	Parity (times)^∗^	GA at admission	GA at delivery	Residence	Educational level	Regular care	Age of patient when CHB diagnosed	Mode of transmission	Acute insults
1	31	2	1	28	29 + 5	Rural	Middle school	No	6 years old	MTCT	Reactivation
2	23	3	1	31 + 2	31 + 2	Rural	Middle school	No	11 years old	MTCT	Reactivation
3	25	1	0	24 + 1	30	Rural	Diploma degree	No	8 years old	Unknown	Reactivation
4	37	3	1	23+	32 + 3	Urban	Middle school	Yes	29 years old	Unknown	Reactivation
5	35	5	1	33 + 2	33 + 3	Urban	Diploma degree	No	22 years old	Unknown	Reactivation

^∗^Excluding the present childbirth. GA: gestational age in weeks; MTCT: mother-to-child transmission; CHB: chronic hepatitis B.

**Table 2:** Clinical manifestations at admission.

Patient	Asthenia	Anorexia	Nausea	Vomiting	Abdominal pain	Abdominal distension	Jaundice	HE
1	+	+	—	—	—	—	+	—
2	+	+	—	—	—	—	+	—
3	+	+	+	+	—	+	+	—
4	+	+	—	—	+	—	+	—
5	+	+	—	—	—	+	+	—

HE: hepatic encephalopathy.

**Table 3:** Laboratory values at admission.

Patient	ALT (U/L)	AST (U/L)	AST/ALT	TBIL (umol/L)	DBIL (umol/L)	GGT (U/L)	ALP (U/L)	ALB (g/L)	PTA (%)	INR	HBV DNA (Lg10)	Cr (umol/L)
1	3596.5	1717.9	0.5	167.9	132.9	50.8	195.5	30	32	2.14	5.13	60
2	374	112.2	0.3	232	162.3	12.5	220.9	22	22	2.96	3.12	51
3	127	72.5	0.6	368.8	297.7	36	110.8	46	39	1.85	6.68	40.2
4	831.9	568.8	0.7	286.6	228.6	33.5	96.6	29.6	33	2.3	7.09	32.5
5	1376.6	1617.2	1.2	133.2	109.2	29.7	143.6	29.6	33.8	1.89	4.45	66
(Mean ± SD)	1260.6 ± 1389.3	817.1 ± 800.7	/	237.7 ± 94.1	186.1 ± 76.8	32.5 ± 13.7	153.5 ± 53.5	31.4 ± 8.8	32 ± 6.2	2.2 ± 0.45	5.3 ± 1.6	50 ± 13.8

ALT: alanine aminotransferase; AST: aspartate aminotransferase; TB: total bilirubin; Cr: creatinine; INR: international normalized ratio; GGT: gamma-glutamyl transpeptidase.

**Table 4:** Treatment.

Patient	HBsAg^	HBeAg^	HbcAb^	GA began antiviral treatment	Antiviral agents	HBV DNA # (lg 10)	HBV DNA## (lg 10)	Special treatment	Hospital day(day)
1	+	+	+	28 + 3	LDT	5.13	2.88	/	39
2	+	+	+	After delivery	ETV	3.12	TND	Transfusion	30
3	+	—	+	20 + 2	LAM	6.68	TND	/	44
4	+	+	+	23 + 6	TDF	7.09	2.56	ALSS	71
5	+	—	+	After delivery	ETV	4.45	TND	Transfusion	17

^: Tested at admission; +: positive; -: negative; HBeAg: hepatitis B e antigen; HBsAg: hepatitis B surface antigen; HbcAb: hepatitis B core antibody; ALSS: artificial liver support system; #: HBV DNA at the start of antiviral treatment; ##: HBV DNA at 2 weeks after antiviral treatment; TND: target not detected (<1.0*E* + 2 IU/mL); ETV: entecavir; LAM: lamivudine; TDF: tenofovir disoproxil; LDT: telbivudine; GA: gestational age in weeks.

**Table 5:** Delivery data and fetal outcomes.

Patient	Mode of delivery	Operative time (min)	Operative blood loss (mL)	Postpartum blood loss (mL)	GA at delivery (weeks)	Anesthesia	Fetus	Fetal birth weight	Newborn outcome
1	CS	40	150	/	29 + 5	Local	SGA	1450 g	Alive
2	CS	45	500	/	31 + 2	Epidural	SGA	1800 g	Alive
3	Induction	/	300	/	30	/	FD	1100 g	/
4	CS	40	200	/	32 + 3	Epidural	SGA	2200 g	Alive
5	CS	35	400	550 mL	33 + 3	Epidural	SGA	2250 g	Alive

CS: cesarean section; FD: fetal demise; SGA: small for gestational age; GA: gestational age in weeks.

**Table 6:** Prognostic scoring system.

Patient	MELD	SOFA	CLIF-SOFA	AARC-ACLF
1	24	4	6	7
2	26	4	7	8
3	25	4	7	9
4	26	4	7	9
5	21	3	6	8

SOFA: sequential organ failure assessment; CLIF-SOFA: chronic liver failure-sequential organ failure assessment; MELD: model for end-stage liver disease; AARC-ACLF: Acute-on-chronic Liver Failure Research Consortium score.

## Data Availability

The main data had been shown in this article. We are committed to the reliability of the data. Due to protect patient privacy, the original data used to support the findings of this study cannot share now and are just available from the corresponding author.

## Abbreviations

DNA: Deoxyribonucleic acid
HBV: Hepatitis B virus
HBV-DNA: Hepatitis B DNA
HBV-ACLF: Hepatitis B virus-related acute-on-chronic liver failure.

## Supplementary Materials

Supplementary Materials Supplementary Table 1. ALT level during hospitalization (U/L).
