# Corrigendum to “Acute-on-Chronic Liver Failure in Pregnant Patients with Chronic Hepatitis B: A Retrospective Observational Case Series Study”

**DOI:** 10.1155/2020/2603491

**Published:** 2020-11-20

**Authors:** Shiwei Wang, Haofeng Xiong, Changling Luo, Hong Zhao, Ying Fan, Ting Zhang, Lili Wang, Qi Wang, Wen Xie

**Affiliations:** ^1^Department of the Liver Center, Beijing Ditan Hospital, Capital Medical University, No. 8 Jingshundong Street, Chaoyang District, Beijing 100015, China; ^2^Department of Critical Care Medicine, Beijing Ditan Hospital, Capital Medical University, No. 8 Jingshundong Street, Chaoyang District, Beijing 100015, China

In the article titled “Acute-on-Chronic Liver Failure in Pregnant Patients with Chronic Hepatitis B: A Retrospective Observational Case Series Study” [1], the acknowledgements should read as follows:

“This article was supported by Beijing Municipal Science and Technology Commission (D171100003117005); Digestive Medical Coordinated Development Center of Beijing Hospitals Authority (XXZ0402); Beijing Hospitals Authority of Hospitals Clinical Medicine Development of Special Funding Support (ZYLX201808).”
