# Supplementary material for: Acute-on-Chronic Liver Failure in Pregnant Patients with Chronic Hepatitis B: A Retrospective Observational Case Series Study
Source: Gastroenterol Res Pract. 2020 Aug 12;2020:9831687. doi: 10.1155/2020/9831687 (PMC7441429; doi:10.1155/2020/9831687)
Supplement: Supplementary Materials — Supplementary Table 1. ALT level during hospitalization (U/L). [file 9831687.f1.pdf]

supplaymentary

Table 1:ALT level during hospitalization(U/L)

Patient	0 week	1th week	2nd week	3rd week	4th week	5th week	6th week	7th week	8th week	9th week	10th week
1	3596.5	138	67.1	30.1	24	20.6	20	/	18.2	/	/
2	374	33.3	73.5	63	28.5	/	/	/	/	/	/
3	127	66.4	75.7	68.7	75.3	75.9	47.1	/	/	/	/
4	831.9	314.3	159.4	54	62.6	42	297.5	221.4	236.8	131.4	78.6
5	1376.6	145.7	42.1	11.7	/	/	/	/	/	/	/

"/":No tests or discharge
